# Evaluation of a single screen and treat strategy to detect asymptomatic malaria among pregnant women from selected health facilities in Lindi region, Tanzania

**DOI:** 10.1186/s12936-020-03513-0

**Published:** 2020-11-30

**Authors:** Chonge Kitojo, Frank Chacky, Emmanuel S. Kigadye, Joseph P. Mugasa, Abdallah Lusasi, Ally Mohamed, Patrick Walker, Erik J. Reaves, Julie R. Gutman, Deus S. Ishengoma

**Affiliations:** 1 US President’s Malaria Initiative, United States Agency for International Development, Dar es Salaam, United Republic of Tanzania; 2 National Malaria Control Programme, Dodoma, United Republic of Tanzania; 3 The Open University of Tanzania, Dar es Salaam, United Republic of Tanzania; 4 USAID Boresha Afya Southern Zone, FHI 360 Dar es Salaam, United Republic of Tanzania; 5 MRC Centre for Global Infectious Disease Analysis, Department of Infectious Disease Epidemiology, Imperial College London, London, UK; 6 Malaria Branch, Division of Parasitic Diseases and Malaria, Center for Global Health, Centers for Disease Control and Prevention and US President’s Malaria Initiative, Dar es Salaam, United Republic of Tanzania; 7 Malaria Branch, Division of Parasitic Diseases and Malaria, Center for Global Health, Centers for Disease Control and Prevention, Atlanta, USA; 8 National Institute for Medical Research, Dar Es Salaam, United Republic of Tanzania; 9 Faculty of Pharmaceutical Sciences, Monash University, Melbourne, Australia; 10 Harvard T.H. Chan School of Public Health, Boston, MA USA

**Keywords:** Malaria in pregnancy, Single screening and treatment for malaria, Antenatal care, Rapid diagnostic teast, Falciparum, Tanzania

## Abstract

**Background:**

In areas of high transmission, malaria in pregnancy (MiP) primarily causes asymptomatic infections; these infections nonetheless increase the risk of adverse maternal and fetal outcomes. In 2014, Tanzania initiated a single screening and treatment (SST) strategy for all pregnant women at their first antenatal care (ANC) visit using malaria rapid diagnostic tests (RDT) for surveillance purposes. However, there is paucity of data on the effectiveness of SST in the prevention of MiP. The objective of this study was to estimate the number of asymptomatic infections among pregnant women detected by SST, which would have been missed in the absence of the policy.

**Methods:**

Data from pregnant women attending their first ANC visits between October 2017 and June 2018, including gestational age, history of fever, and RDT results, were abstracted from ANC registers in eight health centres in two randomly selected districts, Kilwa and Lindi, in Lindi Region. The proportion of symptomatic (with history of fever in the past 48 h) and asymptomatic pregnant women with positive RDTs were calculated and stratified by trimester (first, second and third). The study areas were categorized as low transmission with prevalence < 10% or moderate/high with ≥ 10%.

**Results:**

Over the study period, 1,845 women attended their first ANC visits; 22.1% were in the first trimester (< 12 weeks gestation age). Overall 15.0% of the women had positive RDTs, and there was a trend towards higher malaria prevalence in the first (15.9%) and second (15.2%) trimesters, compared to the third (7.1%), although the differences were not statistically significant (*p* = 0.07). In total, 6.9% of women reported fever within the past 48 h and, of these, 96.1% were RDT positive. For every 100 pregnant women in the moderate/high and low transmission areas, SST identified 60 and 26 pregnant women, respectively, with asymptomatic infections that would have otherwise been missed. Among the 15.9% of women detected in the first trimester, 50.7% were asymptomatic.

**Conclusion:**

In areas of moderate/high transmission, many infected women were asymptomatic, and would have been missed in the absence of SST. The benefits on maternal and fetal birth outcomes of identifying these infections depend heavily on the protection afforded by treatment, which is likely to be greatest for women presenting in the first trimester when intermittent preventive treatment (IPTp) with sulfadoxine-pyrimethamine (SP) is contraindicated, and in areas with high SP resistance, such as most parts of Tanzania. An evaluation of the impact and cost-effectiveness of SST across different transmission strata is warranted.

## Background 

More than 32 million pregnancies occur annually in malaria endemic areas in sub-Saharan Africa [1], with a prevalence of malaria infection during pregnancy ranging from 20 to 35% in the World Health Organization (WHO)-Afro regions [2]. Both symptomatic and asymptomatic malaria infections in pregnancy are associated with increased risk of maternal and fetal complications, including maternal anaemia, spontaneous abortion, low birth weight (LBW; < 2500 g), and maternal death [3–8]. The WHO estimates that in sub-Saharan Africa there were approximately 11 million exposed pregnancies and 872,000 low birth weight infants in 2018 [2].

The United Republic of Tanzania is amongst the highest burden countries, with the third highest estimated malaria deaths in 2018. In mainland Tanzania, 93% of the population live in areas where malaria is transmitted, and approximately 1.7 million pregnant women are at risk of malaria infection annually [9] with a significantly higher burden in the Lake, Western, and Southern zones [10]. The overall prevalence of malaria measured in children 6–59 months declined by 50% between 2015 and 2017 [10, 11], from 14.4 to 7.3%, and ranged from < 1% in the highlands of northern regions to as high as 25% in Western Tanzania [11]. In pregnant women, malaria prevalence declined from 8.1% in 2014 to 6.7% in 2017; with similar heterogeneity as that reported in national surveys among children 6–59 months [12].

The control of MiP involves providing pregnant women with at least 3 monthly doses of sulfadoxine/pyrimethamine (SP) during the second and third trimesters, insecticide-treated nets (ITNs) at first antenatal clinic (ANC) visit, and effective case management with prompt diagnosis and treatment using effective anti-malarials (quinine in the first trimester and artemisinin-based combination therapy [ACT] in the second and third trimesters) [13, 14], following WHO recommendations [15].

To improve surveillance, in 2014 Tanzania introduced a policy of testing of all pregnant women with rapid diagnostic tests (RDTs) for malaria at first ANC visit and treating those who are positive with ACT following WHO recommendations [15], an approach known as single screening and treatment (SST). The SST strategy has the potential to improve the control of MiP by detecting malaria infections among pregnant women and facilitating effective treatment, especially those in the first trimester who are not eligible to receive IPTp and in areas with high SP resistance. SST provides useful and timely data to monitor malaria trends and seasonal variations, to assess heterogeneity in transmission at higher spatial and temporal resolution [12],and to detect areas of increased prevalence in order to target interventions and respond to outbreaks [12, 16]. In addition, these data can be used to monitor exposure of pregnant women to malaria infection [5]. However, diagnosis of malaria in pregnant women can be a challenge, especially in high transmission areas where false negative RDT results may occur as a result of low density parasitaemia or pfhrp2/3 gene deletions [17].

Although SST has been implemented since 2014, there is paucity of data to show its effectiveness in preventing the adverse consequences of MiP, by identifying and treating parasitaemic women who would be missed in its absence. This study evaluated the SST policy in two districts of Lindi region to determine the proportions of symptomatic and asymptomatic women identified by testing to understand its potential benefit, with the asymptomatic women being those who would have been missed without SST.

## Methods

### Study design

**Fig. 1:**
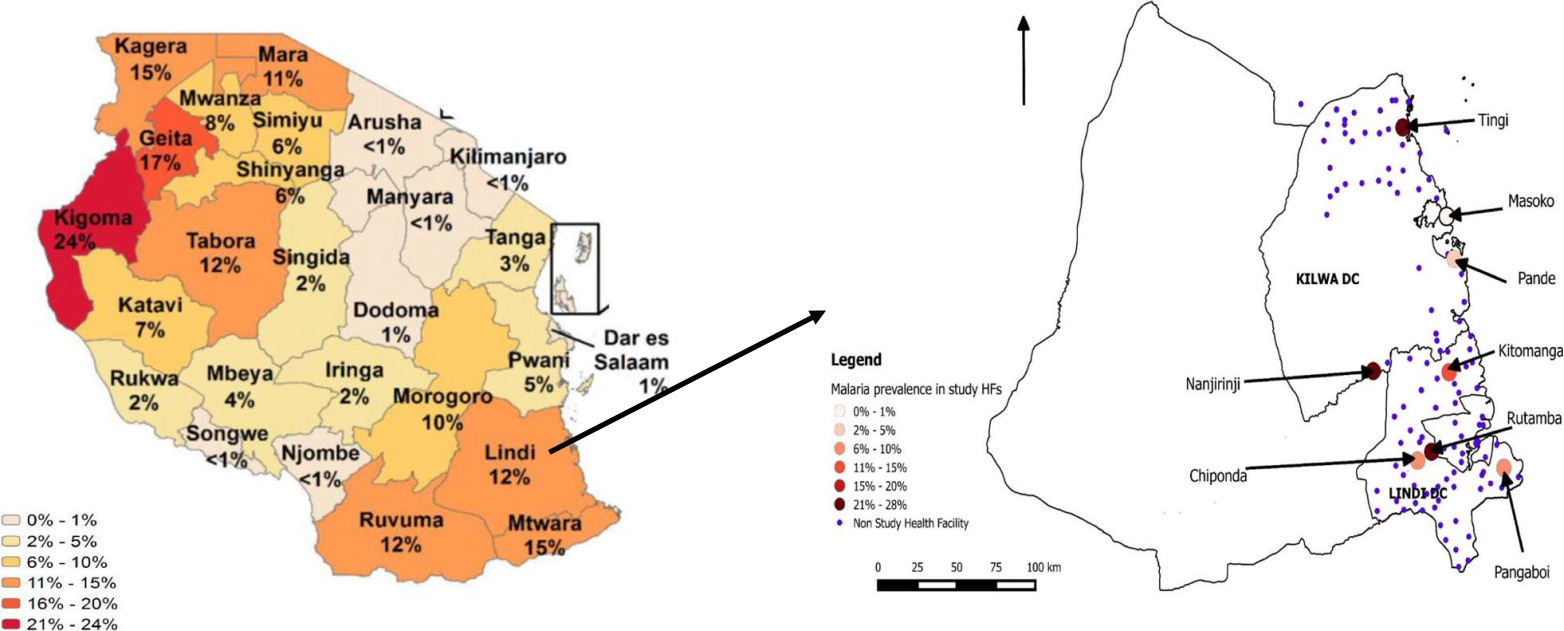
A map of Lindi region showing parasite prevalence in pregnant women attending 1st ANC by health centres included in the study, October 2017–June 2018

This was a descriptive, exploratory, cross-sectional survey that collected data from pregnant women attending their first ANC visits between October 2017 and June 2018 in Kilwa and Lindi districts in Lindi region, southern Tanzania (Fig. 1). Lindi is one of the three regions of Tanzania’s southern zone that experiences bimodal rainfall with peaks in November and April [9], corresponding to the high malaria transmission seasons. The region was purposively selected from among the three regions of Southern zone that have moderate/high malaria transmission, with parasite prevalence in children aged 0–59 months > 12% [10]. The prevalence of malaria in Lindi region was 15% and 12% among children aged 0–59 months in National surveys in 2016 and 2017, respectively [10, 11]. In pregnant women, the prevalence of malaria in Lindi ranged from 13 to 20% between 2014 and 2017 [12].

Kilwa and Lindi districts were randomly selected from among the five districts in Lindi region. In each district, four health centres were then purposively selected from a sampling frame of all 11 health centres in the two districts (73%) based on high volume of ANC attendees, with a minimum of 9 pregnant women per month in Lindi district and 29 women in Kilwa district. The populations of the two districts were similar, 190,744 and 194,143 in Kilwa and Lindi, respectively [18].

### Data analysis

Data were collected using a tool that captured all Health Management Information System (HMIS) indicators, including gestational age in weeks at the time of first ANC attendance and RDT results (SD BIOLINE P.f./Pan; Standard Diagnostic, Suwon City, South Korea), as well as measured fever or history of fever. De-identified data were double-entered into an Excel spreadsheet (Microsoft Office 2010, Seattle WA) by two independent abstractors; discrepancies between the two entries were validated against the register.

Symptomatic malaria was defined as a positive RDT with either fever at presentation (axillary temperature ≥ 37.5^ °^C) or reported history of fever in the last 48 h; asymptomatic malaria infections were defined as a positive RDT without fever. The proportion of symptomatic and asymptomatic pregnant women with malaria who were detected with the SST approach was estimated and stratified by trimester (first, second and third) and malaria transmission (< 10% prevalence defined as low, ≥ 10% as moderate/high). Continuous variables were assessed using *t*-test (for normally distributed variables) or a non-parametric test, such as the Mann–Whitney *U* test (in case the variables were not normally distributed), while Chi-square test was used to compare categorical variables. Logistic regression was used to estimate associations between risk factors for malaria and malaria prevalence after adjusting for location of health centres, fever status, and trimester. Adjusted odds ratios and 95% confidence intervals (CI) are reported. *P*-values < 0.05 were considered statistically significant. Analyses were carried out in STATA version 11.0 (STATA Corp, Texas, and USA). Due to lack of data from DHIS2, the risks of malaria infection among pregnant women of different age groups, gravidity, gestational age, and bed net ownership/use could not be determined because these variables are not normally collected in the routine ANC settings.

### Ethical considerations

Ethical clearance was obtained from the Medical Research Coordinating Committee (MRCC) of the National Institute for Medical Research (NIMR) reference: NIMR/HQ/R.8a/Vol. IX/2713. Additional permission was obtained from the Lindi Regional Medical Officer. Since routine health facility data without personal identifiers were used, no consent was sought from pregnant women per standard practice of the NIMR MRCC.

## Results

### Baseline characteristics

A total of 1845 pregnant women had their first ANC visits between October 2017 and June 2018; 1206 (65.4%) and 639 (34.6%) in Kilwa and Lindi district, respectively. All women who attended their first ANC were tested for malaria by RDT. Overall, 408 (22.1%) were in the first trimester, 1,337 (72.5%) in the second, and 100 (5.4%) were in the third trimester; the mean gestational age was 18.3 weeks (95% CI = 18.0, 18.6). Kilwa district had significantly more women (26.1%) in the first trimester compared to Lindi district (14.6%, *p* < 0.001); the mean gestational age was lower in Kilwa (17.7 weeks, 95% CI = 17.3, 18.1 weeks) compared to Lindi (19.3 weeks; 95% CI = 18.9, 19.8; *p* < 0.001) (Table 1). The average monthly attendance per health facility was higher in Kilwa (mean = 134 women/district/month) compared to Lindi district (mean = 71 women/district /month; *p* < 0.001).

**Table 1:** Characteristics of women stratified by district

District	Health facility	Total pregnant women n (%)	GA in weeks, mean (95% CI*)	Pregnant women with fever, n (%)	Pregnant women with positive RDT, n (%)
Kilwa	Masoko	343 (28.4)	18.3 (17.5–19.1)	4 (1.2)	4 (1.2)
	Nanjilinji	277 (23.0)	18.3 (17.6–19.0)	66 (23.8)	71 (25.6)
	Pande	178 (14.8)	18.0 (17.0–18.9)	8 (4.5)	8 (4.5)
	Tingi	408 (33.8)	16.7 (16.0–17.4)	11 (2.7)	114 (28.0)
	Sub-Total	1206 (65.4)	17.7 (17.3–18.1)	89 (7.4)	197 (16.3)
Lindi	Kitomanga	174 (27.3)	22.3 (21.3–23.3)	15 (8.6)	20 (11.5)
	Pangaboi	193 (30.2)	19.7 (18.9–20.5)	5 (2.6)	11 (5.7)
	Rondo	112 (17.5)	16.5 (15.6–17.3)	6 (5.4)	8 (7.1)
	Rutamba	160 (25.0)	17.6 (16.8–18.4)	12 (7.5)	40 (25.0)
	Sub-Total	639 (34.6)	19.3 (18.9–19.8)	38 (6.0)	79 (12.4)
Total		1845 (100.0)	18.3 (18.0–18.6)	127 (6.9)	276 (15.0)

*95% confidence interval

### Malaria prevalence

**Fig. 2:**
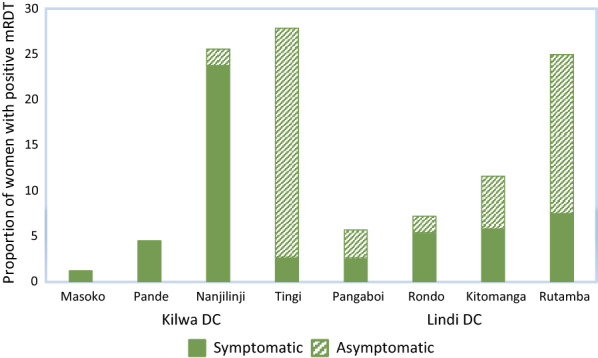
Prevalence of malaria by RDT and proportion of symptomatic and asymptomatic women among those with positive RDTS

Overall, 276 (15.0%) pregnant women had positive RDTs at first ANC visit. There were significant differences in malaria prevalence by district (16.3% vs 12.4% in Kilwa and Lindi, respectively; *p* = 0.02) and substantial heterogeneity among the study facilities, ranging from 1.2 to 27.9% (Fig. 2). Four facilities were classified as being in low transmission settings (prevalence < 10%) and the remaining four facilities were in moderate/high transmission areas (≥ 10%).

In total, 127 (6.9%) women reported fever within the past 48 h. Among symptomatic women (n = 127), all except five (3.9%) had positive RDTs results. Among RDT positive participants, the proportion of asymptomatic infections in the facilities in moderate/high transmission settings were almost twice as high as facilities in low transmission settings (60 vs 26%, Fig. 2).

### Risk factors for malaria infections

Malaria prevalence by RDT was significantly associated with current or recent febrile illness compared to no febrile illness (aOR 5.9, 95% CI = 4.9–6.9) and moderate/high compared to low transmission setting (aOR 2.6, 95% CI = 2.0–3.1; *p* < 0.001). Pregnant women in the first and second trimesters were at higher risk of malaria infection compared to those in the third (15.9, 15.2, and 7.1% prevalence in first, second, and third trimesters, respectively), but the differences were not significant (*p* = 0.07). An increase in gestation age by one week was associated with a reduction in the risk of malaria infection by 6% (aOR = − 0.06, 95% CI = -− 0.1–0.02), *p* = 0.006) (Table 2).

**Table 2:** Risk factors of malaria infections among pregnant women from Kilwa and Lindi Districts

Item		N (%)	Crude OR	*p* value	aOR (95% CI)	*p* value
Fever	No	1718 (93.1)	Ref.		Ref.	
	Yes	127 (6.9)	5.5(4.6–6.4)	< 0.001	5.9 (4.9–6.9)	< 0.001
Trimester	First	408 (22.1)	Ref.		Ref.	
	Second	1338 (72.5%)	− 0.05 (− 0.4–0.3)	0.737	0.8 (0.2–1.4)	0.013
	Third	99(5.4)	− 0.9 (− 1.7–− 0.1)	0.028	0.6 (− 08–2.1)	0.410
Gestational age, weeks	Mean (95% CI)	18.3 (18.0–18.6)	− 0.02 (0.04–0.003)	0.025	− 0.06 (− 0.1–0.02)	0.006
Malaria transmission (location)	Low	826 (44.8)	Ref.		Ref.	
	Moderate	1019 (55.2)	2.09	*P* < 0.001	2.6 (2.0–3.1)	< 0.001

*N* number of pregnant women, *OR* odds ratio, *aOR* adjusted odds ratio, *CI* confidence interval. Adjustments were done for location of health centres, fever status and trimester

## Discussion

This study assessed the utility of SST in detecting asymptomatic malaria infection among pregnant women in areas of varying malaria transmission intensity who would have been missed in the absence of the policy. Sixty percent of infections detected by SST in moderate/high transmission settings were asymptomatic compared to only 26% in low transmission areas. These asymptomatic women tested positive for malaria after presenting for routine screening during their first ANC visit and would not have been detected in the absence of this SST policy. The SST policy was mainly established for surveillance purposes, but it has been recently shown to have other benefits including potential utility in the stratification of malaria [12]. The use of SST to identify and treat asymptomatic and symptomatic pregnant women (irrespective of gestational age) even in areas of very low endemicity as recently shown by Kitojo et al*.* [12] could potentially make it an important tool in ongoing malaria elimination efforts. The risk of malaria was high among women in the first trimester when IPTp is contraindicated. Studies show that these acquired infections are likely to be caused prior to conception [19]. Particularly in the moderate/high transmission areas, a high proportion of these infections were asymptomatic, and, therefore, would likely have remained undetected and untreated in the absence of SST. Although previous studies assessed the benefit of intermittent screening and treatment as an alternative to IPTp-SP [20], no studies have evaluated the added benefit of SST on top of an existing IPTp-SP program. While women presenting in the second or third trimester are given IPTp-SP, which prevents a large proportion of malaria infections [21], given the high levels of parasite resistance to SP in many areas of Tanzania, the impact of treatment with ACT for infections identified through SST in conjunction with IPTp-SP is likely to be greater than the impact of IPTp-SP alone [22]. Furthermore, as IPTp-SP is contraindicated in the first trimester, women presenting in the first trimester stand to benefit greatly from SST, as testing and treatment with recommended anti-malarials would lead to earlier clearance of persistent parasites than would be achieved with IPTp-SP alone, likely leading to better pregnancy outcome [21].

In general, symptoms and complications of malaria are influenced by transmission intensity and individually acquired immunity. In low transmission areas, asymptomatic infections are less common than in high transmission settings, because the population has lower levels of acquired immunity, thus, routine screening is likely to provide less benefit [23]. In areas with moderate/high malaria transmission, such as most parts of Tanzania, many infected people are asymptomatic and hence might not seek treatment [24]. This is also true for multigravid women, who have substantial anti-malarial immunity, including pregnancy specific immunity [5, 25], resulting in low density infections, which may not be easily detected with the current RDTs [26]. These can lead to persistent, asymptomatic *Plasmodium falciparum* infections which might remain undetected and untreated in the absence of testing with a sensitive diagnostic test [13]. Both symptomatic and asymptomatic malaria infection during pregnancy can cause significant problems like maternal anaemia, low birth weight, and stillbirth [4, 25].

In malaria endemic areas, the majority of asymptomatic individuals, including pregnant women, go untreated, encouraging gametocytogenesis, and leading to infectious reservoirs for continued transmission of malaria [17, 27–29]. In addition, children and pregnant women have higher prevalence of malaria with potentially high level of submicroscopic gametocytes compared to non-pregnant adults, highlighting the potential of these groups as a reservoir for transmission [30]. SST may provide a direct benefit to the community, by detecting and treating asymptomatic pregnant women, thus clearing a potential silent reservoir of gametocytes [16]. Thus, strategies to identify and target these infections is important and urgently needed as countries move towards malaria elimination [31].

Kilwa district had significantly more women presenting in the first trimester compared to Lindi district (26.1% vs 14.6%, respectively; *p* < 0.001). The study did not assess the reasons for this pattern; however, these differences could be explained by several factors ranging from cultural beliefs to socio-economic issues and service delivery barriers. In some areas, women will wait for their husbands to give them advice and permission to start ANC, which may delay initiation of care. On the other hand, financial constraints and long distances to the health facilities may also affect early initiation of ANC [32, 33]. Given that delayed access to ANC and late booking have been linked to increased maternal and fetal mortality and morbidity [32–34], these barriers need to be identified and overcome. Early initiation of ANC visits is crucial for pregnant women to access ANC platforms for early detection, prevention and treatment of malaria and other pregnant-related conditions in order to improve maternal and new-born health, resulting in more positive pregnancy outcomes [35]. Given the greater value of SST for women in the first trimester, particularly those from moderate/high transmission areas, that the implementation of SST strategy in addition to IPTp-SP, could by itself help to promote early ANC attendance. However, this needs to be further assessed, and other novel strategies are needed to improve women’s access to early ANC.

This study had several limitations, which might limit the generalizability of the findings. Conceptually, this was an exploratory study and sample size estimation was not based on the prevalence of fever among pregnant women attending their first ANC. As a result, few women (6.9%) were symptomatic, although the majority of these (96.1%) had malaria. This limited the ability to perform in-depth analysis to explore the risk caused by malaria infection to pregnant women of different age, gestation age, gravidity and bed net ownership/use. It is not possible to obtain data on the presence of fever and symptoms from DHIS2, because they are not reported. This study would recommend recording of fever/history of fever and other symptoms of malaria among ANC attendees to provide more robust numbers to assess the proportion of asymptomatic women who are identified as a result of screening at the first ANC. In addition, data on women’s age, gravidity and bed net ownership/use were not available, thus it was not possible to assess the differential sensitivity of SST stratified by these variables. It is likely that the potential benefits of SST for individual women decrease with increasing gravidity, but this will need to be further assessed in future studies. Additionally, in moderate/high transmission areas, older women tend to be less susceptible to malaria infection than younger women, due to higher levels of immunity [5]. Thus, future studies should assess the effects of gravidity and maternal age on the effectiveness of SST. Furthermore, RDTs were used to detect malaria at ANC; but they have been reported to have low sensitivity for detecting infections with low-density parasitaemia, suggesting that the number of asymptomatic infections might have been underestimated, potentially warranting comparative testing with more sensitive tests such as PCR.

## Conclusion

In areas of moderate/high transmission, the majority of pregnant women with RDT detectable infections were asymptomatic, thus only identifiable through routine testing such as SST. In the context of IPTp-SP, the benefit of identifying these infections depends heavily on the effectiveness of IPTp-SP, which depends on the level of SP resistance. Although SST can potentially have a substantial benefit in the first trimester when IPTp-SP is contraindicated, even women later in their pregnancy can still benefit from testing and treatment with an ACT over SP, given the level of SP resistance in most parts of Tanzania. In lower transmission areas, a higher proportion of the RDT positive women were symptomatic, likely reflecting lower levels of immunity, though a quarter of infected women would still not have been detected through case management alone. The impact of treatment with an ACT in conjunction with IPTp-SP likely benefits pregnant women and improves pregnancy outcomes. Further evaluation of the impact of SST in conjunction with IPTp-SP on birth outcomes and the cost-effectiveness of this approach across different transmission strata is warranted.

## Data Availability

Data from this study can be accessed upon request though corresponding author who will submit a formal request to the Ministry of Health through the National Malaria Control Programme.

## Abbreviations

ANC: Antenatal Care
IPTp: Intermittent Preventive Treatment in pregnancy
ITNs: Insecticide-treated mosquito nets
LLIN: Long-lasting insecticide-treated nets
MiP: Malaria in Pregnancy
NMCP: National Malaria Control Programme
NIMR: National Institute for Medical Research
SP: Sulfadoxine-Pyrimethamine
SST: Single Screening and Treatment
WHO: World Health Organization
